# Evaluation of the Effects of Tetrahydrocannabinol (THC) and Cannabidiol (CBD) on Gingival and Skin Keratinocyte Growth, Migration, Metabolic Activity, and Pro-Inflammatory Cytokine Secretion

**DOI:** 10.3390/biomedicines13102541

**Published:** 2025-10-17

**Authors:** Maryam Bahraminia, Fatima-Zahrae Laaboudi, Charlotte Romanet, Ze Zhang, Jamila Chakir, François Béland, Mahmoud Rouabhia

**Affiliations:** 1 Groupe de Recherche en Écologie Buccale, Faculté de Médecine Dentaire, Université Laval, Quebec City, QC G1V 0A6, Canada; maryam.bahraminia.1@ulaval.ca (M.B.); fatima-zahrae.laaboudi.1@ulaval.ca (F.-Z.L.); 2 Axe Médecine Régénératrice Centre de Recherche du CHU de Québec—Université Laval, Département de Chirurgie de la Faculté de Médecine, Université Laval, Quebec City, QC G1V 0A6, Canada; ze.zhang@fmed.ulaval.ca; 3 Institut Universitaire de Cardiologie et de Pneumologie de Québec, Université Laval, Quebec City, QC G1V 0A6, Canada; charlotte.romanet.1@ulaval.ca (C.R.); jamila.chakir@fmed.ulaval.ca (J.C.); 4 SiliCycle, Inc., 2500 Bd du Parc Technologique, Quebec City, QC G1P 4S6, Canada; francoisbeland@silicycle.com

**Keywords:** CBD, THC, keratinocytes, anti-inflammatory diseases, cytokines, drug delivery

## Abstract

**Background**: Cannabinoids, such as tetrahydrocannabinol (∆-9-THC) and cannabidiol (CBD) have been proposed for topical medicinal use as a treatment for tissue inflammation. In this context, keratinocytes are the first cells that encounter cannabinoids. The present study evaluated the dose–response relationship between different concentrations of THC and CBD and their effects on human skin and gingival keratinocyte growth and migration, to identify suitable non-toxic concentrations of cannabinoids. **Methods:** Human gingival and skin keratinocytes were exposed to CBD or THC at different concentrations for 24 h, and then cell adhesion, morphology, and growth/viability were assessed. The effects of cannabinoids on keratinocyte migration were evaluated at 6, 12, and 24 h. Cytotoxicity of CBD and THC against keratinocyte cells was assessed using an LHD cytotoxicity test. Cell metabolic profiles were evaluated using Mito and Glyco Stress Assays. The anti-inflammatory effects of cannabis derivatives were assessed against LPS-stimulated keratinocytes. Data analysis was performed by one-way ANOVA. **Results:** Only high concentrations (10 and 20 μg/mL) of CBD and THC were cytotoxic to gingival and skin keratinocytes, reduced cell adhesion and growth, and were associated with a delay in cell migration after wounding. Cells exposed to high concentrations (20 μg/mL) of cannabinoids displayed high levels of lactate dehydrogenase (LDH) activity and changes in mitochondrial activities. CBD induced a metabolic shift in skin keratinocyte cells toward glycolysis, while reducing mitochondrial oxidative phosphorylation. In contrast, THC did not alter the metabolic profile of skin keratinocytes. Interestingly, both CBD and THC significantly reduced the LPS-induced inflammatory response by decreasing secretion of IL-6 and IL-8 by gingival and skin keratinocytes. **Conclusions:** Gingival and skin keratinocytes interact differently with cannabinoids. Only high concentrations of cannabinoids were cytotoxic, suggesting that the use of low concentrations of CBD and THC for topical medicinal applications may help control tissue inflammation.

## 1. Introduction

Cannabis is a substance derived from the cannabis plant, which consists of three species: Cannabis *sativa*, Cannabis *indica*, and Cannabis *ruderalis*. Although three species of Cannabis exist, Cannabis *sativa* is most commonly used for medicinal purposes. Cannabis *sativa* contains hundreds of bioactive constituents, including phytocannabinoids, a group of C21 or C22 containing terpenophenolic compounds [1,2]. The predominant cannabinoids extracted from the cannabis plant are delta-9-tetrahydrocannabinol (THC), cannabidiol (CBD), and cannabinol (CBN), followed by cannabigerol (CBG), cannabichromene (CBC), and cannabinodiol (CBND) [3]. Medicinally, cannabis is used to relieve symptoms such as pain, fever, anxiety, and diarrhea in the context of numerous diseases [4]. Furthermore, cannabis products (THC and CBD) have been reported to possess anti-inflammatory activity and have been proposed for use in the treatment of inflammatory diseases [5]. THC mimics the active site binding properties of an endogenous cannabinoid, anandamide, while CBD mimics the 2-arachidonoyl glycerol [6,7]. Because THC is a partial agonist of cannabinoid receptor CB1, and CB2 [8], numerous studies have investigated its therapeutic potential [9]. Indeed, it has been demonstrated that THC and its synthetic analogs have anti-inflammatory activity in different cells and tissues [10]. For example, THC can activate CB1 receptors to promote the migration of periodontal ligament fibroblasts, by enhancing adhesion between cells and the extracellular matrix [11]. CBD also exhibits numerous beneficial pharmacological effects, including anti-inflammatory and antioxidant properties [12]. CBD was successfully delivered transdermally in different species, to evaluate its anti-inflammatory activity [13]. Thus, the two major derivatives of the cannabis plant, THC and CBD, could have potential for medicinal use in the control of tissue inflammation.

Inflammatory disorders of skin and oral mucosa tissues are often accompanied by pruritus and pain, which are associated with negative psychological effects and represent a substantial burden to patients. Moreover, tissue inflammation can lead to permanent scarring, further exacerbating this burden [14]. Cannabinoids have been reported to exert their anti-inflammatory effects by modulating inflammatory mediators and cellular functions [15]. In support of this, cannabinoids have been shown to decrease the secretion of multiple inflammatory cytokines by various cells [16]. Cannabis products can be administered to the human body through various routes. Although smoking is most frequently associated with cannabis use [17], for medicinal purposes, cannabis smoke could be potentially damaging to patients, because it contains toxic components that have been shown to reduce cell viability [18]. Medicinal cannabis products can be effectively delivered through transepithelial-dermal (oral mucosa, skin) routes. Such delivery methods could represent an easy and efficient route for the treatment of localized skin and oral mucosa inflammation. Topical application of THC or CBD oil to skin and gingival mucosa brings these products directly into contact with epithelial (gingival tissue) and epidermal (skin tissue) keratinocytes. Indeed, keratinocytes have immunological functions that eliminate pathogens and promote tissue repair. As such, the involvement of THC and CBD in reducing tissue inflammation could be due to their interactions with skin keratinocytes and gingival epithelial cells. However, few studies have investigated the dose effects of THC and CBD on the behaviors of skin and gingival keratinocytes. The present study evaluated the effects of THC and CBD on the viability and growth of gingival and skin keratinocytes, as well as their effects on cell migration and wound healing. The effects of THC and CBD on the mitochondrial energetic profiles of these cells, and the release of pro-inflammatory cytokines were also investigated.

## 2. Materials and Methods

### 2.1. Reagents

Dulbecco’s modified Eagle’s medium with Ham’s F-12 (DMEH) and fetal bovine serum (FBS) were obtained from Thermo Fisher Scientific (Quebec, QC, Canada).

Penicillin/Streptomycin (P/S), fungizone (F), 3-(4,5-dimethylthiazol-2-yl)-2,5-diphenyl-tetrazolium-bromide) (MTT), phenol red, glucose, epidermal growth factor (EGF), EDTA-free protease inhibitor cocktail, protease inhibitor, phosphatase inhibitor cocktail, lipopolysaccharides (LPS) from *Escherichia coli* O111:B4, and the lactate dehydrogenase (LDH) cytotoxicity detection kit were purchased from Millipore (Millipore Sigma, ON, Canada). IL-6 ELISA kit, IL-8 ELISA kit, and Normocin^®^ were purchased from Cedarlane Canada (Burlington, ON, Canada). Sodium pyruvate (Cat-103578-100), and L-glutamine (Cat-103579-100) were purchased from Agilent Technologies (Santa Clara, CA, USA). Protein extraction lysis buffer was obtained from Cell Signaling (Danvers, MA, USA, Cat-9803).

### 2.2. Cells and Cell Culture Steps

In the present study, “skin cells” refer to human HaCat keratinocytes (Cat-T0020001, Cedarlane, Burlington, ON, Canada), a type of spontaneously transformed aneuploid immortal skin keratinocyte cell line [19]. Gingival epithelial cells refer to a non-cancerous human gingival epithelial cell line, GMSM-K [20], kindly provided by Dr. Daniel Grenier (Laval University, Québec City, QC, Canada). Skin and gingival keratinocytes were cultured in Dulbecco’s modified Eagle’s medium with Ham’s F-12 (DMEH) supplemented with 5 µg/mL human transferrin, 2 × 10^−9^ M 3,3′,5′-triiodo-L-thyronine, 0.4 µg/mL hydrocortisone, 10 ng/mL epidermal growth factor (EGF), 100 IU/mL penicillin G, Normocin^®^ at 50 µg/mL, and 10% fetal bovine serum. The medium was changed three times per week. When the culture reached 80% confluence, cells were detached by enzymatic treatment, washed twice, counted, and adjusted to a final concentration of 10^6^ cells/mL for subsequent use. For experiments, cells were used between passage 5 and 10. Routine testing for mycoplasma was performed each week on the cells by Hoechst staining [21].

### 2.3. Cannabis Derivatives

High-purity CBD and THC were extracted from *Cannabis sativa* plants by SiliCycle, Inc. (Quebec, QC, Canada). Extraction of CBD and THC from hemp biomass was conducted as described previously [22]. CBD and THC isolates were solubilized in methanol to yield a 3 mg/mL stock solution, and used at various concentrations (2, 4, 6, 8, 10, and 20 µg/mL) corresponding to approximately 6 to 60 × 10^−3^ µM: these concentrations were selected based on previously published studies [22,23,24,25,26].

### 2.4. Effects of CBD and THC on Keratinocyte Morphology

Keratinocytes (skin and gingival cells) were seeded (2 × 10^5^ cells/well), in duplicate, in 6-well cell culture plates and cultured in a 5% CO_2_ incubator at 37 °C. The medium was changed every 24 h until the culture reached 80% confluence. At this time, the medium was refreshed and supplemented with either CBD (2, 4, 6, 8, 10 or 20 μg/mL), THC (2, 4, 6, 8, 10 or 20 μg/mL) or vehicle (negative control). CBD and THC were added from 3 mg/mL stock solutions in methanol; thus, as a negative control, cells were cultured in the presence of an equivalent amount of methanol (0.66%, *v*/*v*). At this level, methanol was not cytotoxic, as previously reported [22]. Cells in the presence or absence of CBD or THC were cultured for 24 h in a 5% CO_2_ atmosphere at 37 °C. Adherent cells in each well were observed under an inverted optical microscope, using a 20x objective (Nikon Canada, Mississauga, ON, Canada). Images of all conditions (control, CBD and THC exposed cells) were recorded using a Coolpix 950 camera (Nikon Canada), as previously reported [27]. Cell morphology in CBD and THC treated cells were compared to non-treated cells. Four biological replicates (n = 5) were performed.

### 2.5. Effects of CBD and THC on Keratinocyte Cell Viability and Growth

Keratinocytes were seeded in duplicate at a density of 2 × 10^5^ cells/well in 6-well cell culture plates. Cells were incubated in a 5% CO_2_ incubator at 37 °C, and cell culture medium was changed every 24 h until cells reached 80% confluence. Resulting cell cultures were then exposed for 24 h to one of the following concentrations of CBD or THC: 2, 4, 6, 8, 10, or 20 μg/mL, in 10% FBS-supplemented medium. For a negative control, cells were cultured in the presence of 0.66% methanol (*v*/*v*). After this step, each well was washed twice with fresh culture medium to remove any remaining CBD or THC left in the culture medium. Then, cells were supplemented with 1 mL culture medium and subjected to an MTT colorimetric staining assay [28,29]. Following addition of MTT, cell cultures were incubated for 3 h at 37 °C. Supernatant was then removed, and the cell cultures were washed twice with phosphate-buffered saline (PBS). Following the last wash, 1 mL of 0.04 N HCl-isopropanol was added to each well, and cell cultures were incubated with for 15 min at room temperature with shaking. Each culture condition was distributed in the wells of a 96-well plate, and a 200 μL solution containing dissolved formazan was added to each well (in triplicate). Absorbance was read at 570 nm using an X-Mark microplate spectrophotometer (Bio-Rad, Mississauga, ON, Canada). Six biological replicates were performed (n = 6).

### 2.6. Effects of CBD and THC on Cell Migration

Skin and gingival keratinocytes were seeded (3 × 10^5^ cells/well) in duplicate, in 6-well plates and cultured in a 5% CO_2_ atmosphere at 37 °C. The medium was refreshed every 24 h until the cultures reached 100% confluence, as determined by inverted optical microscope observations. The cell layer in each well was then cross-scratched horizontally and vertically using a 200 µL sterile micropipette tip held perpendicular to the bottom of each well. The generated wound was approximately 0.44 to 0.50 mm in width [29]. Each well was washed twice with warm culture medium to remove the cell suspension generated by the scratch. Then, 2 mL of medium containing either CBD or THC at one of the following concentrations (2, 4, 6, 8, 10, or 20 μg/mL), or a solvent-only negative control, were added to each well and incubated at 37 °C in a 5% CO_2_ humid atmosphere. As a negative control, cells were cultured in the presence of 0.66% methanol (*v*/*v*). Cell migration through the edge of each wound was recorded using an inverted optical microscope, with a 4X objective (Nikon Canada), at 6, 12, and 24 h post-wound and photographed using a digital camera (Coolpix 950). Four photos were taken from each culture condition, one from each cross-scratch wound zone. Photos at different times of incubation were taken from the same cross-scratch zone. Wound closure was determined by measuring the distance between the opposite edges of a wound as a function of time, using a public domain NIH ImageJ image processing program, version 1.48v). Results are presented as the percentage of the initial wound (distance at time zero) using the following formula: ((distance at initial scratch − distance after an identified culture period)/(distance at initial scratch)) × 100%, as previously reported [29]. Five biological replicates (n = 5) were performed. CBD-stimulated, THC-stimulated or unstimulated (vehicle-only control) cell cultures were compared, and the difference was considered significant at *p* < 0.05.

### 2.7. Effect of CBD and THC on Keratinocyte Lactate Dehydrogenase Activity

Skin and gingival keratinocytes were seeded in duplicate at 3 × 10^5^ cells/well in 6-well plates. Cells were cultured in a 5% CO_2_ incubator at 37 °C for 3 days, and medium was changed every 24 h. Cells were then exposed to either CBD or THC at 2, 4, 6, 8, 10, or 20 μg/mL, or vehicle (negative control) in 2 mL of culture medium and incubated for an additional 24 h. For the negative control, cells were cultured in the presence of vehicle, 0.66% methanol (*v*/*v*). At the end of cell culture, the culture supernatant was collected from each well, clarified by centrifugation to remove debris, and then used to evaluate lactate dehydrogenase (LDH) activity via an LDH cytotoxicity assay [30]. LDH is a stable cytoplasmic enzyme found in all cells. It is rapidly released into the cell culture supernatant when the plasma membrane is damaged following contact with stress agents [31]. Contact with CBD or THC chemicals could lead to cell stress, causing LDH release. For this purpose, 50 µL of each collected supernatant was mixed with 50 µL of reconstituted substrate mix in the wells of 96-well plates, followed by a 30 min incubation in a dark atmosphere at 22 °C. At the end of this incubation period, each well was supplemented with 50 µL of an acid solution. Then the absorbance of each well was measured at 490 nm using an X-Mark microplate spectrophotometer (Bio-Rad). As a positive control (PC), corresponding to maximum LDH release, skin and gingival keratinocytes were incubated in the presence of 1% Triton X-100. As a negative control (NC), cells were cultured in culture medium without any stimulation. Cytotoxicity (%) was calculated using the following formula: LDH activity release = ((Cannabinoid exposed cell_absorbance_ − NC_absorbance_)/(PC_absorbance_ − NC_absorbance_)) × 100%. Five biological replicates (n = 5) were performed.

### 2.8. Effect of CBD and THC on Mitochondrial Oxygen Consumption and the Rate of Extracellular Acidification

The oxygen consumption rate (OCR) and basal extracellular acidification rate (ECAR) of skin and gingival keratinocytes were measured using a Seahorse Bioscience XFe24 Extracellular Flux Analyzer (Agilent Technologies, Santa Clara, CA, USA), as previously reported [32]. Skin cells were plated at 6 × 10^5^ cells/well, and gingival cells were plated at 3 × 10^5^ cells/well in 24-well Seahorse cell culture plates. Both cell types were then cultured for 24 h in a 5% CO_2_ atmosphere at 37 °C, until they reached approximately 80% confluence. Then, cells were exposed to 2, 6, or 10 μg/mL of CBD or THC for 24 h. As a negative control (vehicle), the same cell types were cultured in 24-well Seahorse cell culture plate, and were exposed only to culture media with 0.33% methanol (*v*/*v*). Each condition was tested in triplicate. Cell-free wells containing only assay medium were used for background correction. After exposure to CBD or THC, cells were examined under a microscope to confirm cell adhesion, morphology, and uniformity of the cell monolayer before being subjected to Mito- and Glyco Stress Assays.

#### 2.8.1. The Mito Stress Assay to Evaluate OCR

Skin and gingival cell cultures were washed twice with 800 µL /well of Mito Stress Medium (MSM). MSM consists of a mixture of 70 mL of XF base medium (Agilent Technologies, Cat-103335-100) supplemented with 3 mg/L of Phenol Red, 1.75 mL of 1 M glucose, 700 µL of 100 mM sodium pyruvate, and 1.4 mL of 200 mM L-glutamine, with an adjusted pH of 7.4. Then, 525 µL of MSM were added to each well and incubated for 1 h at 37 °C without CO_2_. After this incubation, the OCR was established by sequentially exposing cells to 4 μM oligomycin, 4 μM FCCP (carbonyl cyanide-p-trifluoromethoxy-phenyl-hydrazone), and a combination of the mitochondrial complex I inhibitor rotenone and the mitochondrial complex III inhibitor antimycin A at 8 μM.

#### 2.8.2. The Glyco Stress Assay to Evaluate ECAR

Cell cultures were washed twice with 800 µL/well of glycolysis stress media (GSM). GSM medium is composed of a mixture of 70 mL of XF base medium supplemented with 3 mg/L of phenol red and 700 µL of 200 mM L-glutamate, pH 7.4. After washing, 525 µL of GSM was added to each well and incubated for 1 h at 37 °C in the absence of CO_2_. After this period of incubation, the ECAR was established by sequential injection of 80 mM glucose, 4 μM oligomycin, and 62.5 mM 2-deoxy-D-glucose, a competitive glucose inhibitor (Millipore-Aldrich, Oakville, ON, Canada).

For both Mito and GSM assays, Seahorse cell culture plates were loaded into an XF 24-well microplate reader; and OCR or ECAR values were assessed using the following setup: 3 cycles of 3 min mix, 2 min wait, and 3 min measure.

After completion of each assay, raw OCR and ECAR data were normalized to the concentration of total cellular protein in each corresponding well, which was measured using a Bradford assay. Briefly, adherent cells were washed with PBS and lysed using 15 µL of protein extraction lysis buffer supplemented with an EDTA-free protease inhibitor cocktail, a protease inhibitor, and a phosphatase inhibitor cocktail. The protein concentration in each well was then determined based on a bovine serum albumin (BSA) standard curve. Seahorse Wave Desktop Software, version v2.6 (Agilent Technologies) was used to link measured protein concentrations with the appropriate corresponding well, via the software’s normalization function, providing a simple method for applying the normalization process to the collected raw data (OCR, ECAR). Moreover, background correction was applied using cell-free wells, and background subtraction was performed using Wave software v2.6, ensuring that the metabolic signal measured is from cells. Results were expressed as the percentage change in OCR or ECAR over baseline, defined as the last measurement before the first injection, which was set as 100%, as previously reported [33]. Metabolic parameters were calculated according to the manufacturer’s instructions (Agilent Technologies), and the effects of different concentrations of CBD or THC were normalized to results obtained for control cells. Control cells were cultured in the presence of 0.33% methanol (*v*/*v*)), and not treated with CBD or THC. Data are expressed as the mean ± SD of three biological replicates (n = 3).

### 2.9. Effects of CBD and THC on IL-6 and IL-8 Secretion by Keratinocytes Following LPS Stimulation

Keratinocytes were seeded at 2 × 10^5^ cells/well in 6-well cell culture plates and incubated in a 5% CO_2_ incubator at 37 °C, with medium changes every 24 h until cells reached 60% confluence. Cell cultures were then exposed for 24 h to 2, 4, or 6, μg/mL of CBD or THC, in the presence or absence of 5 μg/mL of bacterial lipopolysaccharide (LPS) [34]. As a negative control, cells were cultured in the presence of 0.2% methanol (*v*/*v*). Culture medium was collected from each condition and used to quantitatively determine IL-6 and IL-8 secretion by gingival and skin keratinocytes, using the sandwich enzyme-linked immunosorbent assay, with three wells for each culture supernatant [35]. ELISA plates were read at 450 nm using a Microplate Reader Model 680 (Bio-Rad). The minimum detectable concentrations were reported by the manufacturer to be less than 0.7 pg/mL for IL-6 and 1 pg/mL for IL-8. Data are expressed as the mean ± SD of three biological replicates (n = 3).

### 2.10. Statistical Analyses

Experiments were done with various biological replicates. Data are expressed as the mean ± SD. The statistical significance of differences between results from control experiments (conducted in the absence of cannabis derivatives) and test experiments (conducted in the presence of CBD or THC) was determined by two-way ANOVA. Posteriori comparisons were performed using Tukey’s method. Normality and variance assumptions were verified using the Shapiro–Wilk test and the Brown and Forsythe test, respectively. Seahorse data were expressed as the mean ± standard error of the mean (SEM). The normality of the data distribution was assessed using a Shapiro–Wilk test. Data normally distributed were analyzed by one-way ANOVA followed by Dunnett’s post hoc tests. Conversely, Kruskal–Wallis tests, followed by Dunn’s post hoc analysis, were used for non-normally distributed data. Statistical significance was defined as a *p*-value < 0.05.

## 3. Results

### 3.1. Exposure to CBD or THC Did Not Affect Keratinocyte Morphology

**Figure 1:**
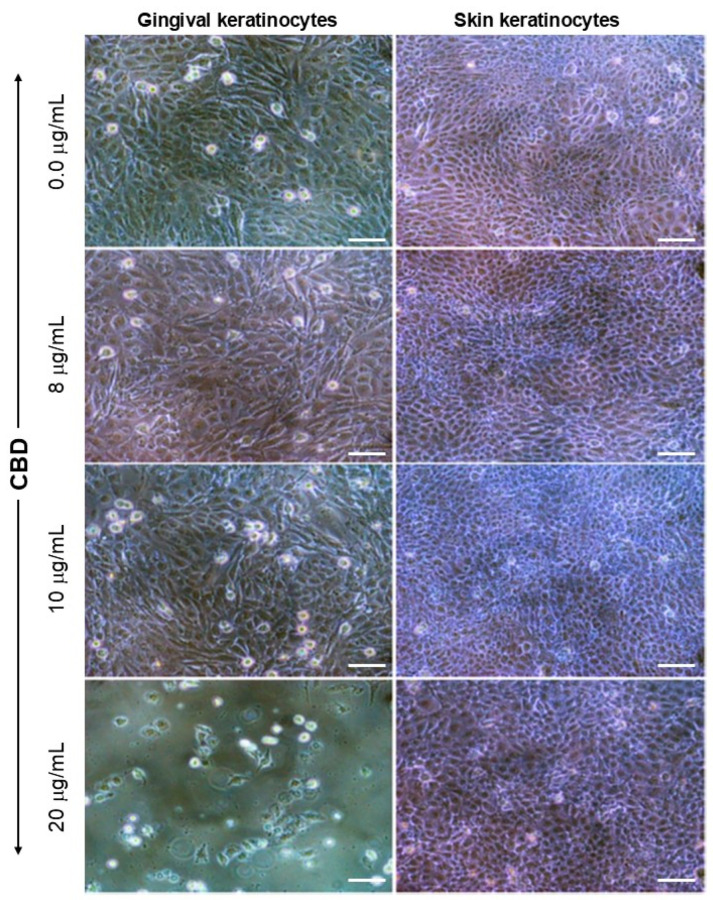
CBD affects cell morphology and adhesion properties in human keratinocytes. Gingival and skin keratinocytes were exposed to various concentrations of CBD for 24 h. Adherent cells were observed and photographed under an optical microscope. Representative photos out of six biological replicates (n = 6) are presented. Scale bar = 50 μm. CBD-non treated cells refer to control cells cultured in the presence of 0.66% methanol (*v*/*v*).

After 24 h of exposure to CBD, keratinocyte morphology was evaluated using an optical microscope. As shown in Figure 1, the morphology of gingival cells was observed to change only at high concentrations of CBD. Indeed, as can be seen in Figure 1, cells in the control group exhibited small, cuboidal shapes, characterized by a high cell density. Following exposure to CBD, no morphological changes were observed with different concentrations, including 8 and 10 µg/mL (Figure 1). However, at 20 µg/mL of CBD, gingival epithelial cells displayed a round morphology, with limited elongated forms (Figure 1). For skin keratinocytes, observations with an optical microscope showed limited morphological changes (Figure 1). Indeed, Figure 1 shows that non-treated skin keratinocytes have a small size and cuboidal shape, with a high cell density of adherent skin keratinocytes. Exposure to CBD at various concentrations resulted in a cell morphology that was similar to control (non-exposed) cell cultures (Figure 1).

**Figure 2:**
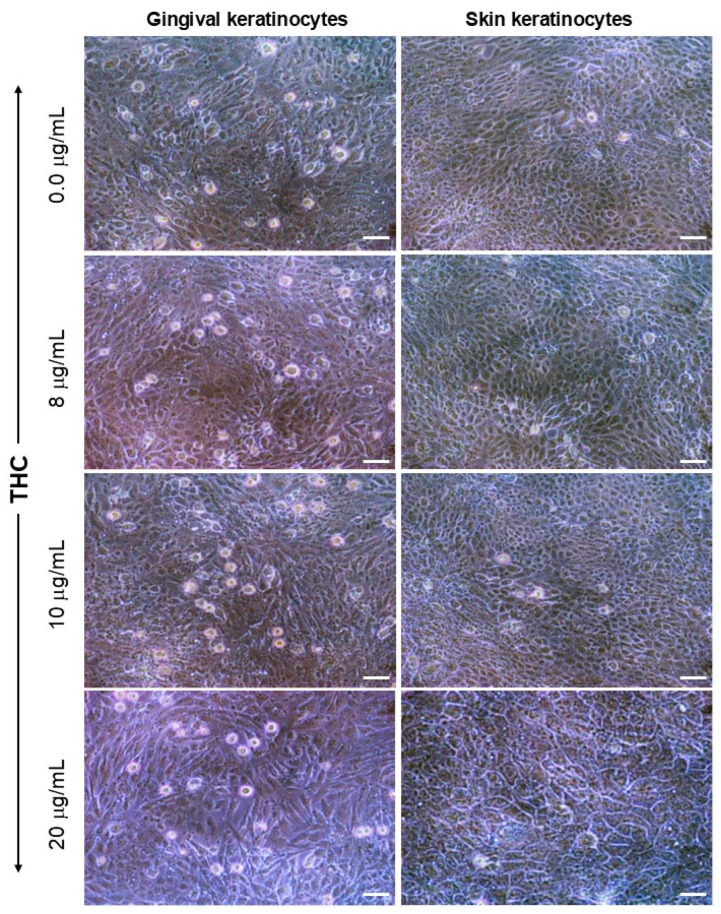
THC affects cell morphology and adhesion properties in human keratinocytes. Gingival and skin keratinocytes were exposed to various concentrations of THC for 24 h. Adherent cells were observed and photographed under an optical microscope. Representative photos out of six biological replicates (n = 6) were presented. Scale bar = 50 μm. THC-non treated cells refer to control cells cultured in the presence of 0.66% methanol (*v*/*v*).

Using a second set of gingival and skin keratinocyte cultures, the effect of THC on cell morphology was evaluated. As shown in Figure 2, gingival keratinocytes exhibited small sizes with high adherent cell density in non-exposed (control) cultures. Similar observations were obtained upon exposure to THC concentrations up to 10 µg/mL (Figure 2). However, at a concentration of 20 µg/mL THC, several gingival keratinocytes exhibited slightly larger sizes, with reduced cell density (Figure 2). The morphology of skin keratinocytes and gingival keratinocytes were similar after exposure to THC (Figure 2). Indeed, for skin keratinocytes, the cell size and density were comparable between non-THC-exposed and THC-exposed cells at various tested concentrations up to 10 µg/mL (Figure 2). However, skin keratinocytes exposed to 20 µg/mL of THC were larger in size than control cells and displayed a reduced cell density (Figure 2). Overall, these results indicate that THC at concentrations ranging from 2 to 10 µg/mL has no adverse effects on the morphology of gingival and skin keratinocytes. At 20 µg/mL, both gingival and skin keratinocytes displayed morphological changes, becoming larger in size, with reduced cell density.

### 3.2. Exposure to CBD or THC Slightly Affects Cell Viability in Keratinocytes

**Figure 3:**
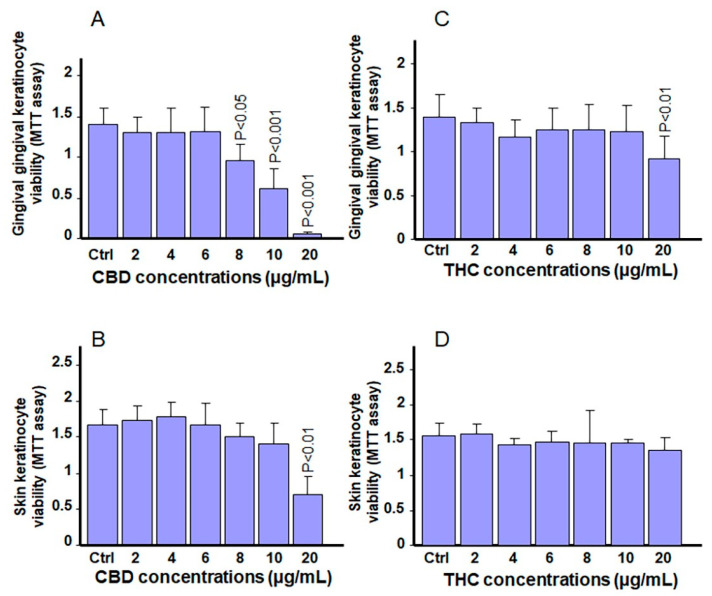
CBD and THC differentially modulate keratinocyte cell viability and growth. Gingival and skin keratinocytes were stimulated with various concentrations of CBD or THC for 24 h. They were then subjected to an MTT assay to evaluate cell viability and growth, by measuring absorbance at 570 nm. (**A**) gingival cells with CBD, (**B**) skin cells with CBD, (**C**) gingival cells with THC, and (**D**) skin cells with THC. *p*-values were obtained by comparing CBD- or THC-stimulated cells to control cells (0.66% methanol (*v*/*v*)). Statistical significance was defined as *p*-values < 0.05, using a two-way ANOVA, n = 5.

To confirm the above described cell morphology results, the effects of CBD or THC on keratinocyte cell viability were evaluated using an MTT assay. As shown in Figure 3A, no significant decrease in the viability of gingival cells was found after 24 h of culture in the presence of CBD, at lower concentrations ranging from 2 to 6 µg/mL. However, at mid-to-high CBD concentrations ranging from 8 to 20 µg/mL, a significant (*p* < 0.001) decrease in cell viability was observed; both compared to control cells and cells treated with lower CBD concentrations (2 to 6 µg/ mL). Although gingival keratinocytes appear to be more sensitive to higher concentrations of CBD, similar results were not obtained for skin keratinocyte cells. As shown in Figure 3B, the cell viability of skin keratinocytes treated with CBD at 2 to 10 µg/ mL was comparable to that of control (non-treated) cells. Only exposure to 20 µg/ mL of CBD led to a significant (*p* < 0.001) decrease in cell viability in skin keratinocytes. The effect of THC on cell viability was also evaluated (Figure 3C,D). Based on an MTT assay, significant (*p* > 0.05) differences in gingival cell viability were only observed upon exposure to 20 µg/ mL THC (Figure 3C). In contrast, for skin keratinocytes, no tested concentrations of THC resulted in decreased cell viability (Figure 3D). Taken together, these results demonstrate that gingival cells are more sensitive than skin keratinocytes to mid and high concentrations of CBD or THC. In contrast, skin keratinocytes were only sensitive to high concentrations of CBD, and tolerated even high concentrations of THC.

### 3.3. Low and Mid Concentrations of CBD and THC Have No Adverse Effects on Cell Migration

**Figure 4:**
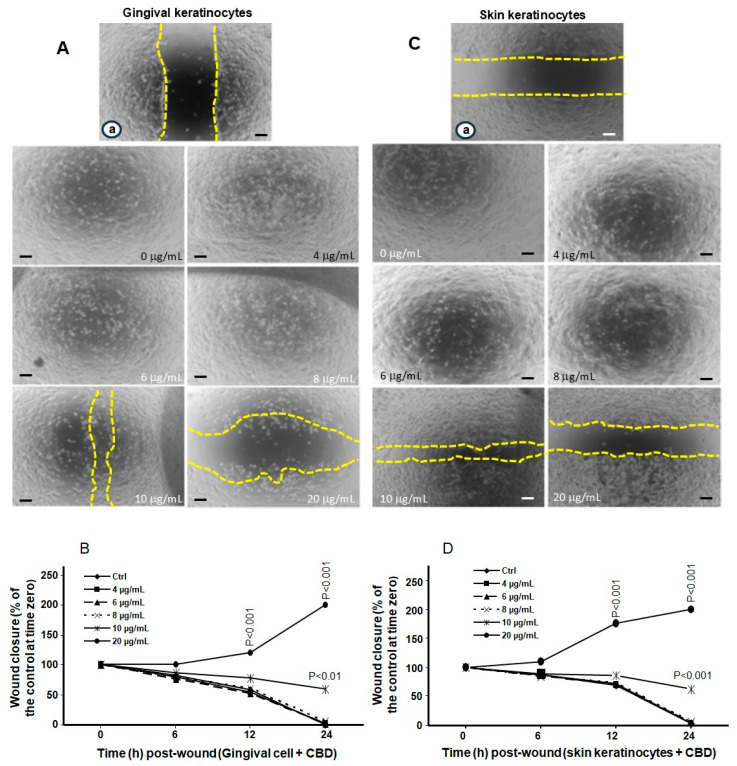
Keratinocyte cell migration is only delayed at high concentrations of CBD. Gingival and skin keratinocytes were cultured until they reached confluence. Cell layers were wounded and exposed to CBD at various concentrations. Cell migration was monitored for 24 h using an optical microscope and photographed. Yellow dashes showed the wound edges. Panels (**A**) (gingival keratinocytes) and (**C**) (skin keratinocytes) show images (Scale bar = 50 μm) at time zero (a) and 24 h post-wounding in the presence of absence of CBD stimulation. Panels (**B**,**D**) display quantitative measurements of cell migration at various time points. *p*-values were obtained by comparing results from CBD or THC treated cells to control cells (0.66% methanol (*v*/*v*)). Statistical significance was defined as a *p*-value < 0.05, using a two-way ANOVA, n = 5.

Gingival and skin keratinocytes must undergo cell migration as part of the wound healing process. To evaluate the effects of CBD and THC on cell migration, a scratch test was used. Cell migration was assessed over 24 h while cells were exposed to either CBD or THC. Figure 4A shows microscope images of gingival cell migration in control cultures vs. those exposed to different concentrations of CBD. Gingival keratinocytes exposed to 4, 6, and 8 µg/mL of CBD showed similar cell migration properties as control cells (Figure 4A) after 24 h of culture. At 10 µg/mL of CBD, cell migration was somewhat inhibited vs. controls; while at 20 µg/mL of CBD, cell migration was inhibited, with cell detachment leading to a more enlarged wound area (Figure 4A) compared to at time zero (Figure 4A, a). These observations by microscope were confirmed by quantitative measurements of wound closure at different times post-wound. As can be seen in Figure 4B, cell migration between control cells and cultures treated with 4, 6, and 8 µg/mL of CBD were comparable. At 10 µg/mL CBD, the cells did migrate, but did not reduce the wound space completely. At 20 µg/mL CBD, there was a significant (*p* < 0.001) increase in the distance separating the two wound edges, which was approximately two times that measured at time zero (Figure 4B).

Monitoring skin keratinocyte migration after 24 h exposure to various concentrations of CBD revealed (Figure 4C,D) a delay in wound healing at higher concentrations (10 and 20 µg/ mL). Skin keratinocyte cells exposed to 4, 6, and 8 μg/mL of CBD, or control cells, showed similar cell migration capabilities, and filled wound space (Figure 4C). However, upon exposure to 10 and 20 µg/mL of CBD, there were still uncovered spaces in the wound area at 24 h. It should be noted that there were less uncovered spaces in cultures treated with 10 μg/mL of CBD than for those treated with 20 μg/mL (Figure 4C). Thus, there is a delay in skin keratinocyte migration following cell exposure to higher concentrations of CBD. These qualitative results were confirmed by quantitative measurements of the space separating cell edges at 6, 12, and 24 h, which corresponds to cell migration capacity. Figure 4D showed that skin keratinocytes treated with lower concentrations (4, 6, and 8 µg/mL) of CBD exhibited the same cell migration levels as control (non-treated) cells. However, there was a slight migration delay in the presence of 10 µg/mL of CBD, and a substantial portion of the wound area remained uncovered following cell exposure to 20 µg/mL of CBD. Altogether, these cell migration results demonstrate that lower concentrations of CBD have no adverse effects on the cell migration capabilities of gingival and skin keratinocytes after wound formation. However, at higher doses (10 and 20 µg/mL), cell migration was delayed; and this delay was greater with gingival compared to skin keratinocyte cells.

**Figure 5:**
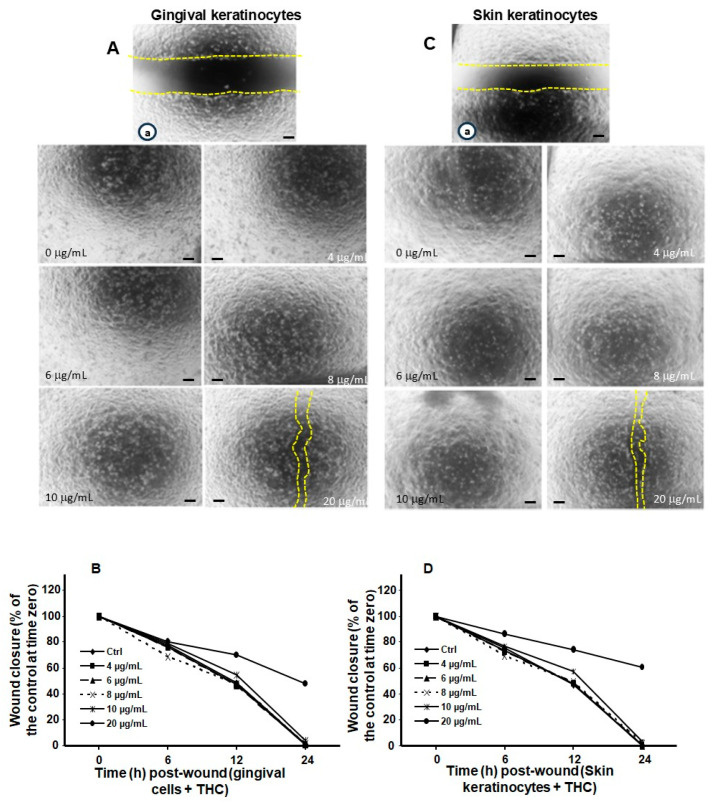
Treatment of keratinocytes with high concentrations of THC slightly delays cell migration. Gingival and skin keratinocyte cells were cultured until they reached confluence. Cell layers were wounded and exposed to THC at various concentrations. Cell migration was followed for 24 h using an optical microscope and photographed. Yellow dashes showed the wound edges. Panels (**A**,**C**) show photos (Scale bar = 50 μm) at time zero (a) and 24 h post-wounding in the presence of absence of THC treatment. Panels (**B**,**D**) display quantitative measurements of cell migration at various time points. Statistical significance was defined as *p*-value < 0.05, using two-way ANOVA, n = 5.

The effects of THC on cell migration were also evaluated using a monolayer cell scratch test. Figure 5A shows that gingival cells displayed a migration delay, compared to control cells, only upon treatment with 20 µg/mL of THC. Quantitative measurements confirmed the lack of adverse effects from THC treatment on gingival cell migration at concentrations ranging from 2 to 10 µg/mL (Figure 5B). However, at 20 µg/mL, THC exposure was associated with a significant delay in cell migration. Comparable results were obtained with skin keratinocytes exposed to THC (Figure 5C,D). Observations under a microscope revealed (Figure 5C) uncovered wound areas in skin keratinocyte cultures exposed to 20 µg/mL of THC (Figure 5C). At lower concentrations, all cultures exhibited similar cell migration, and covered the entire wound space. These observations were confirmed by quantitative measurements of the distance separating wound edges at different times (Figure 5D). Based on this analysis, continuous cell migration occurred up to 24 h post-wound, leading to wound closure in cultures exposed to 4, 6, 8 and 10 µg/mL of THC compared to the control. However, in the presence of 20 µg/mL of THC, there was a significant (*p* < 0.001) delay in skin keratinocyte cell migration (Figure 5D). Altogether, cell migration analysis showed that CBD exposure at higher doses (10 and 20 µg/mL) led to a greater delay in cell migration than THC at the same concentrations. Interestingly, at concentrations of 8 µg/mL or less, neither cannabinoid had an adverse effect on the migration of gingival and skin keratinocyte cells. To confirm these effects on cell migration, LDH activity was assayed in cells treated with CBD, THC or vehicle (control cells).

### 3.4. Low and Mid-Range Concentrations of Cannabinoids Were Not Cytotoxic to Gingival and Skin Keratinocytes

**Figure 6:**
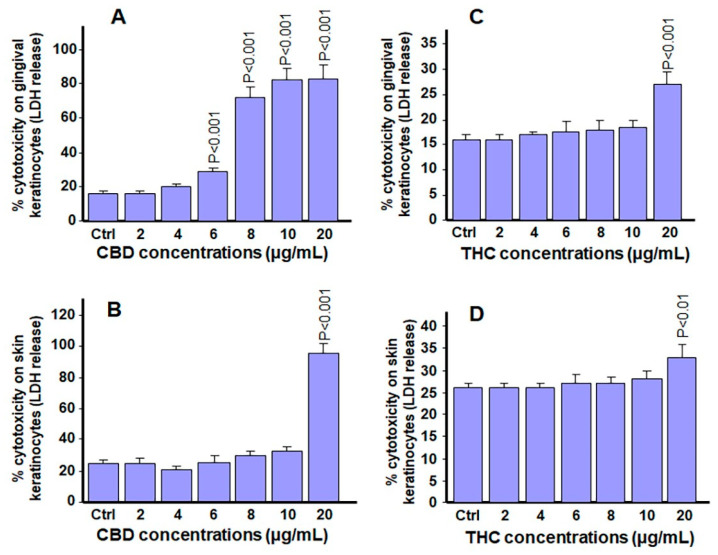
Effect of cannabinoids on LDH activity in keratinocyte cells. Cells were cultured for 24 h and then exposed to CBD or THC for 24 h. Supernatants were collected and analyzed using an LDH assay. (**A**) gingival cells with CBD, (**B**) skin cells with CBD, (**C**) gingival cells with THC, and (**D**) skin cells with THC. *p*-values were obtained by comparing CBD or THC treated cells to control cells treated only with 0.66% methanol (*v*/*v*). Statistical significance was defined as *p*-value < 0.05, using a two-way ANOVA, n = 4.

Because a decrease in cell viability and cell migration was observed after exposure to higher concentrations of either CBD or THC, we hypothesized that these effects could be associated with a corresponding increase in LDH activity under the same conditions. As shown in Figure 6A, there was an increase in LDH activity following exposure of gingival keratinocytes to CBD. Such an increase in LDH activity was observed starting from 6 µg/mL and continued through 8, 10, and 20 µg/mL. In contrast, with skin keratinocytes only exposure to 20 µg/mL of CBD resulted in a high level of LDH activity (Figure 6B).

Based on these results, gingival keratinocytes are more sensitive than skin keratinocytes to CBD exposure at high concentrations. Using a similar set of experiments, LDH activity was also analyzed following exposure of gingival and skin keratinocytes to THC. Figure 6C showed that only exposure to a high concentration (20 µg/mL) of THC induced a significant (*p* < 0.001) increase in LDH activity in gingival keratinocytes. All other concentrations of THC showed LDH activities that were similar to controls. Similar results were obtained for skin keratinocytes (Figure 6D). To investigate further the safety or cytotoxicity of the tested cannabinoids on gingival and skin keratinocytes, Seahorse analysis was performed to assess the mitochondrial activity of the cells.

### 3.5. CBD and THC Exposure Does Not Induce Adverse Effects on Oxygen Consumption or Extracellular Acidification Rates in Gingival Keratinocytes

**Figure 7:**
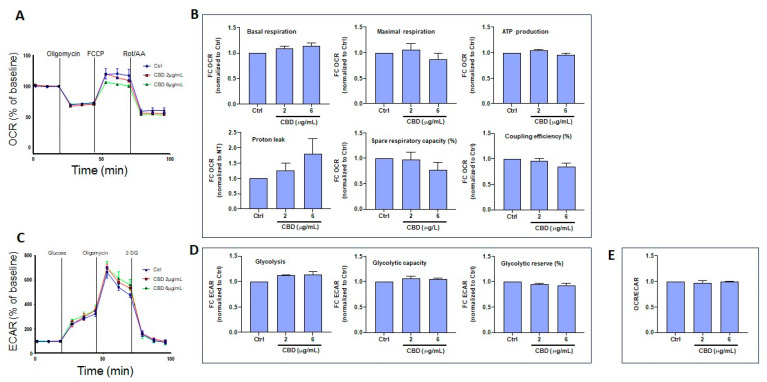
Effect of CBD on the energetic profile of gingival keratinocytes. (**A**) Representative curves from Seahorse measurements of the oxygen consumption rates (OCR) of epithelial gingival cells following exposure to CBD. (**B**) Quantification of basal respiration, maximal respiration, ATP production, proton leak, spare respiratory capacity, and coupling efficiency in epithelial gingival cells (n = 3). (**C**) Representative curves for Seahorse measurements of extracellular consumption acidification rates (ECAR) in epithelial gingival cells following exposure to CBD. (**D**) Quantification of individual parameters: glycolysis, glycolytic capacity, and glycolytic reserve in epithelial gingival cells (n = 3), and (**E**) OCR/ECAR ratio. Statistical significance was defined as *p*-value < 0.05, using a one-way ANOVA, n = 3.

Based on a set of experiments, Seahorse bioenergetics analyses were conducted to quantify oxygen consumption rates (OCR) in gingival keratinocytes exposed to CBD (Figure 7A). The collected results are presented in Figure 7B, showing that CBD at low (2 μg/mL) and intermediate (6 μg/mL) concentrations did not affect basal or maximal respiration, ATP production, proton leak, spare respiratory capacity (SRC), or coupling efficiency (CE) compared to controls (Ctrl). CBD also did not affect extracellular acidification rates (ECAR) in gingival keratinocytes (Figure 7C). Indeed, after exposure of gingival cells to CBD, no changes in glycolysis, glycolytic capacity, or glycolytic reserve were recorded (Figure 7D). Moreover, the OCR/ECAR ratio was similar between CBD-exposed and control gingival keratinocytes (Figure 7E). Results from ORC and ECAR analyses indicate that low and intermediate CBD concentrations do not affect mitochondrial function or glycolysis in gingival keratinocytes. At a higher dose (10 μg/mL), CBD was toxic to gingival keratinocytes, making ORC and ECAR determinations impossible.

**Figure 8:**
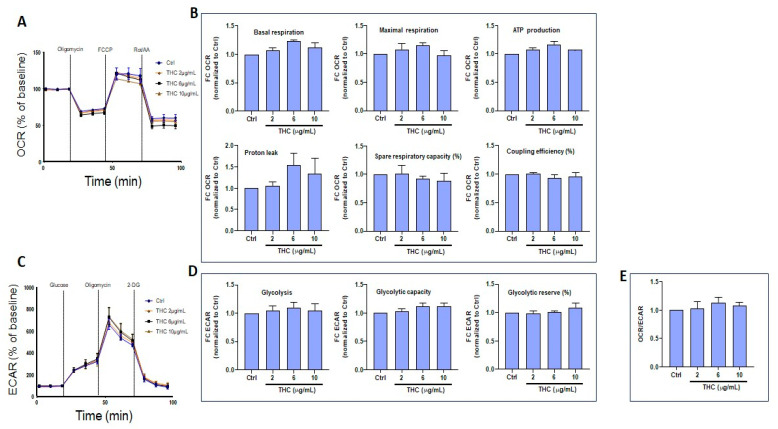
Effect of THC on the energetic profile of gingival keratinocyte cells. (**A**) Representative curves for Seahorse measurements of OCR in epithelial gingival cells exposed to THC. (**B**) Quantification of individual parameters for basal respiration, maximal respiration, ATP production, proton leak, spare respiratory capacity, and coupling efficiency in epithelial gingival cells treated as indicated. (**C**) Representative curves for Seahorse measurements of ECAR in epithelial gingival cells treated with 2, 6, or 10 µg/mL THC vs. control cells. (**D**) Quantification of individual parameters of glycolysis, glycolytic capacity, and glycolytic reserve in gingival cells treated as indicated, and (**E**) OCR/ECAR ratio. Statistical significance was defined as *p*-value < 0.05, using a one-way ANOVA, n = 3.

Similar experiments were performed to measure the effects of THC exposure at different concentrations (2, 6, and 10 μg/mL); and no adverse effects were observed for gingival keratinocytes. Namely, both OCR (Figure 8A,B) and ECAR (Figure 8C,D) were found to be unchanged compared to controls. It is interesting to note that, even at concentrations as high as 10 μg/mL, THC was not toxic to gingival keratinocytes. The OCR/ECAR ratio was similar between THC-exposed and control gingival keratinocyte cells (Figure 8E).

### 3.6. CBD and THC Treatment Modulates Oxygen Consumption and Extracellular Acidification Rates in Skin Keratinocytes

**Figure 9:**
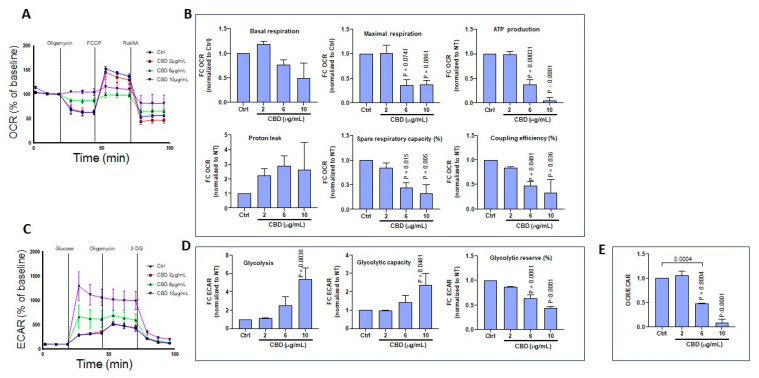
Effect of CBD on the energetic profile of skin keratinocyte cells. (**A**) Representative curves from Seahorse measurements of OCR in skin keratinocytes exposed to CBD. (**B**) Quantification of individual parameters for basal respiration, maximal respiration, ATP production, proton leak, spare respiratory capacity, and coupling efficiency in skin keratinocyte cells treated as indicated. (**C**) Representative curves from Seahorse measurements of ECAR in skin keratinocytes treated with 2, 6, or 10 µg/mL CBD vs. control cells. (**D**) Quantification of individual parameters of glycolysis, glycolytic capacity, and glycolytic reserves in skin keratinocyte cells treated as indicated, and (**E**) OCR/ECAR ratio. Statistical significance was defined as *p*-value < 0.05, using a one-way ANOVA, n = 3.

**Figure 10:**
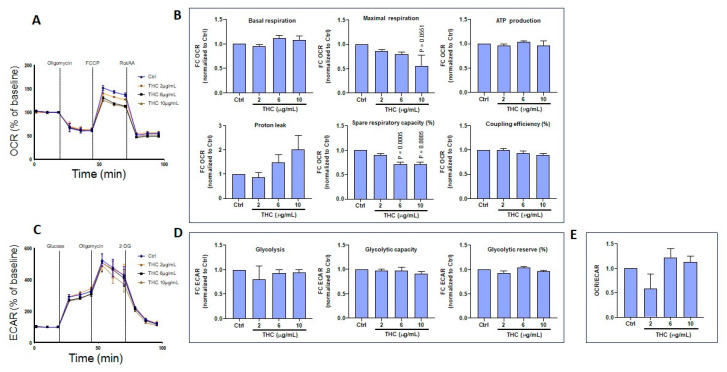
Effect of THC on the energetic profile of skin keratinocyte cells. (**A**) Representative curves from Seahorse measurements of OCR in skin keratinocytes treated with 2, 6, or 10 µg/mL THC vs. control cells. (**B**) Quantification of individual parameters for basal respiration, maximal respiration, ATP production, proton leak, spare respiratory capacity, and coupling efficiency in skin keratinocyte cells treated as indicated. (**C**) Representative curves from Seahorse measurements of ECAR in skin keratinocytes treated with 2, 6, or 10 µg/mL THC vs. control cells. (**D**) Quantification of individual parameters of glycolysis, glycolytic capacity and glycolytic reserve in skin keratinocyte cells treated as indicated, and (**E**) OCR/ECAR ratio. Statistical significance was defined as *p*-value < 0.05, using a one-way ANOVA, n = 3.

Using another set of experiments, Seahorse bioenergetics analyses were performed to quantify OCR in skin keratinocytes exposed to CBD (Figure 9A). Based on these results lower OCRs were observed following exposure to higher concentrations of CBD (Figure 9B). Indeed, at 10 μg/mL, CBD tended to decrease basal respiratory rates. Furthermore, CBD treatment at 6 and 10 μg/mL reduced maximal respiration, ATP production, spare respiratory capacity and coupling efficiency (Figure 9B). However, CBD did not affect proton leak (Figure 9B). Overall, these findings demonstrate that CBD at intermediate (6 μg/mL) and higher (10 μg/mL) doses can induce mitochondrial dysfunction in skin keratinocytes. By contrast, glycolysis assessment revealed an increase in ECAR at intermediate and higher doses of CBD (Figure 9C). Metabolic analysis showed that CBD at 10 μg/mL increased glycolysis and glycolytic capacity in skin keratinocytes (Figure 9D). However, CBD significantly (*p* = 0.001) reduced glycolytic reserves in a dose-dependent manner (Figure 9D). These observations are consistent with a decreased OCR/ECAR ratio (Figure 9E). Similarly, the effect of THC on energetic metabolism in skin keratinocytes was investigated, revealing that THC affected the OCR profile (Figure 10A) in these cells. THC treatments at higher concentrations (10 μg/mL) tended to decrease maximal respiration, as well as spare respiratory capacity (Figure 10B). However, exposure to THC did not alter the ECAR profile (Figure 10C). Also, THC did not modify glycolysis, glycolytic capacity or glycolytic reserves (Figure 10D). In agreement with these results, the OCR/ECAR ratio between THC-exposed and control cells remained unchanged (Figure 10E). In conclusion, CBD induces a metabolic shift in skin keratinocyte cells toward glycolysis, while decreasing mitochondrial oxidative phosphorylation, whereas THC does not change the metabolic profile of skin keratinocytes.

### 3.7. CBD and THC Decrease LPS-Induced Secretion of IL-6 and IL-8 by Gingival and Skin Keratinocytes

**Figure 11:**
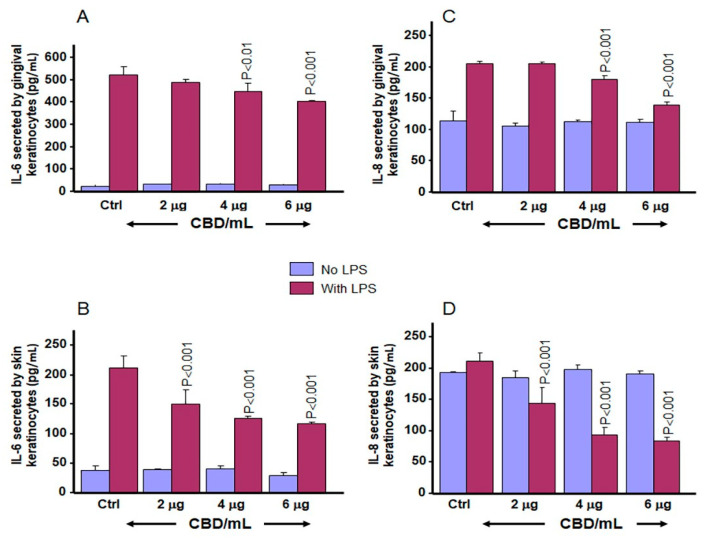
Effect of CBD on IL-6 and IL-8 secretion by gingival and skin keratinocytes. Cells were stimulated with LPS (5 µg/mL) or not (unstimulated control) in the presence or absence of CBD at various concentrations for 24 h. The culture supernatant from each condition was collected and subjected to an ELISA assay to quantify levels of IL-6 (**A**,**B**), and IL-8 (**C**,**D**) secretion. Data are expressed as the mean ± SD. Statistical significance was defined as *p*-value < 0.05, using a two-way ANOVA, n = 3.

To investigate the anti-inflammatory effects of CBD and THC, keratinocyte cells were stimulated with LPS at 5 µg/mL in the presence or absence of CBD or THC at concentrations of 2, 4, and 6 µg/mL for 24 h. As shown in Figure 11, basal levels of IL-6 secreted by gingival and skin keratinocytes were low and did not exceed 30 pg/mL. Addition of CBD at various concentrations did not increase IL-6 secretion in keratinocytes. LPS stimulation resulted in a significant increase in IL-6 secretion for gingival keratinocytes, from 24 ± 5 pg/mL in control cells to 524 ± 34 pg/mL in LPS treated cells (Figure 11A). Interestingly, the presence of CBD significantly (*p* < 0.01) decreased secretion of IL-6 by gingival keratinocytes (Figure 11B). With skin keratinocytes, IL-6 levels increased from 38 ± 8 pg/mL with control cells to 212 ± 21 pg/mL following LPS stimulation (Figure 11B). However, this LPS-induced increase in IL-6 secretion by skin keratinocytes was significantly (*p* < 0.01) decreased by CBD treatment (Figure 11B).

The effect of CBD on IL-8 secretion was also investigated; and a significant (*p* < 0.001) decrease in IL-8 was measured after LPS stimulation. As shown in Figure 11C,D, stable basal secretion of IL-8 was seen even in conjunction with CBD treatment. Addition of LPS at 5 µg/mL led to a significant increase in IL-8 secretion by gingival (Figure 11C) and skin (Figure 11D) keratinocytes compared to control, unstimulated keratinocytes. However, this increase in IL-8 secretion under LPS stimulation was decreased when CBD was added simultaneously with LPS (Figure 11C,D).

**Figure 12:**
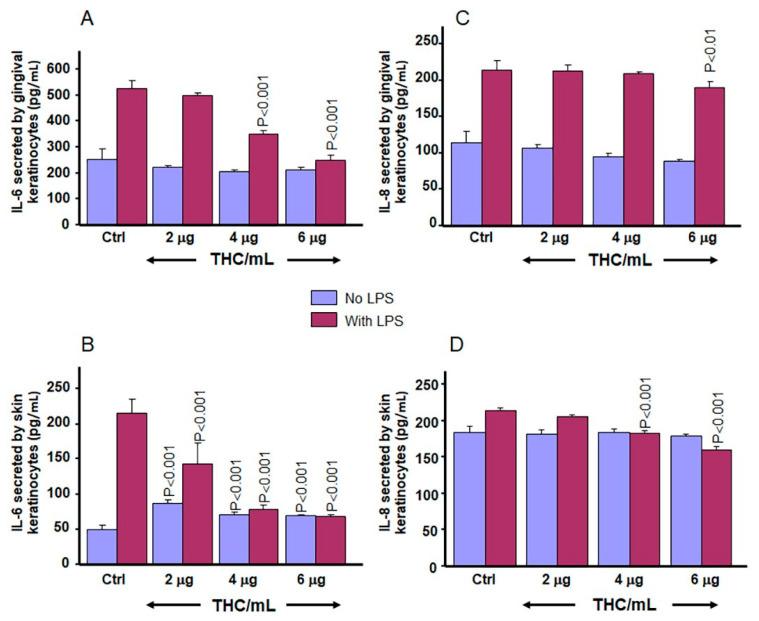
Effect of THC on IL-6 and IL-8 secretion by gingival and skin keratinocytes. Cells were stimulated with LPS (5 µg/mL) or not (unstimulated control) in the presence or absence of THC at various concentrations for 24 h. The culture supernatant from each condition was collected and subjected to an ELISA assay to quantify levels of IL-6 (**A**,**B**) and IL-8 (**C**,**D**) secretions. Data are expressed as the mean ± SD. Statistical significance was defined as *p*-value < 0.05, using a two-way ANOVA, n = 3.

Using another set of experiments, IL-6 and IL-8 secretions were measured in gingival and skin keratinocytes stimulated with or without LPS at 5 µg/ mL in the presence of various concentrations of THC. These results show (Figure 12) a decrease in LPS-induced IL-6 secretions in keratinocytes after exposure to THC. In addition, THC slightly but significantly (*p* < 0.01) reduced LPS-induced IL-8 secretion in gingival and skin keratinocytes (Figure 12C,D). Altogether, these results demonstrate that CBD and THC may exert their anti-inflammatory effects in part by reducing secretion of pro-inflammatory mediators by tissue keratinocytes.

## 4. Discussion

Phytocannabinoids, including CBD and THC, are well-known for their beneficial effects for the treatment of skin disorders such as acne, dry skin, eczema, psoriasis, and periodontal inflammation [36,37,38]. Cannabinoids can modulate keratinocyte activity by controlling inflammation and promoting or inhibiting wound healing [39]. The present study demonstrates that use of CBD and THC at concentrations comparable to or below those used previously for clinical [40,41], preclinical [23] and experimental [24,25] applications, are not cytotoxic to skin and gingival keratinocyte cells. Moreover, at these concentrations, CBD and THC even stimulate cell growth slightly; while high concentrations of CBD and THC can reduce keratinocyte growth. These results confirm those previously reported for oral cancer cell lines [42,43]. The present study reveals that CBD and THC have a dose-dependent impact on human gingival and skin keratinocytes. Such an observation confirms previously reported results with skin keratinocytes [43,44]. Altogether, the present study demonstrates that low concentrations of CBD and THC cannabinoids are not cytotoxic to gingival and skin keratinocytes. However, at high concentrations, THC, and even more so CBD, may be harmful to keratinocytes. Thus, to promote skin health, cannabinoids should be used at concentrations below 10 µg/mL. This observation is confirmed by the present study, which evaluated the effects of CBD and THC on cell migration during wound recovery. Similar keratinocyte migration activities were observed between control cells and those exposed to low and moderate concentrations of CBD or THC, as previously reported [43,45]. It is interesting to note that high concentrations of CBD inhibited keratinocyte cell migration to a greater extent than high concentrations of THC. These observations are in accordance with those previously published [46], in an investigation of CBD and THC oil for the treatment of chronic pressure ulcers. In that study, the authors reported that daily administration of CBD and THC oil to patients led to improved healing of pressure ulcers in 2 weeks and complete closure after 2 months. The high concentration of cannabinoids that caused cytotoxic effects in gingival and skin keratinocytes also caused an increase in the levels of LDH activity. Specifically, treatment of cells with a high concentration (20 µg/mL) of CBD or THC was associated with a high level of LDH activity in both gingival and skin keratinocytes. However, importantly, at lower concentrations (2 to 8 µg/mL), LDH activity was comparable to that of controls. Moreover, even at a moderately high concentration of 10 µg/mL, only an insignificant increase in LHD activity was observed. These results are in agreement with those previously reported, showing that increased LDH activity is only associated with high concentrations of cannabinoids [47,48].

Cytotoxicity is often associated with changes in mitochondrial activity. In the present study, CBD treatment at 2 and 4 μg/mL did not affect cell respiration, ATP production, proton leak, spare respiratory capacity, or coupling efficiency. However, at a higher concentration of 10 µg/mL, CBD treatment was associated with deregulation of mitochondrial activity in gingival keratinocytes. Thus, CBD at low concentrations is unlikely to affect cell mitochondrial activity. However, use of CBD at high concentrations could affect mitochondrial activity [49]. On the other hand, THC can be tolerated at both low and high concentrations (10 µg/mL) in gingival keratinocytes [50]. The present study suggests that gingival keratinocytes are more sensitive to CBD than to THC. Treatment of cells with either CBD or THC at 10 µg/mL tended to decrease keratinocyte mitochondrial activity, as previously reported [51]. Overall, based on these results, CBD treatment induces a metabolic shift in skin keratinocytes toward glycolysis, while decreasing mitochondrial oxidative phosphorylation. In contrast, THC treatment does not alter the metabolic profile of skin keratinocytes.

Cannabis derivatives, including CBD and THC, were reported to control inflammatory diseases [34,39]. Because of this, the present study evaluated the effects of low concentrations of CBD and THC on LPS-induced inflammation in gingival and skin keratinocytes: neither CBD nor THC was found to promote secretion of pro-inflammatory cytokines (IL-6 and IL-8). However, CBD at the tested concentrations significantly decreased LPS-induced IL-6 and IL-8 release in gingival and skin keratinocytes. These results are comparable to those reported for keratinocyte cells stimulated with *Cutibacterium acnes*-derived extracellular vesicles (CEVs) before treatment with CBD at 0.5, 1 and 2 μm; where decreased secretion of IL-6, IL-8 and TNFα was reported [52]. The effects of THC on the secretion of pro-inflammatory cytokines by gingival and skin keratinocytes were slightly different compared to those obtained with CBD. Namely, there was a significant decrease in IL-6 secretion by gingival and skin keratinocytes exposed to LPS in the presence of THC. These results are in agreement with a report that THC exerts its anti-inflammatory effects by reducing IL-6 secretion in human monocytes stimulated with TLR agonists [25]. Measurements of IL-8 secretion following LPS stimulation in gingival and skin keratinocytes show that THC treatment results in only a slight decrease in IL-8 secretion. This observation confirms previous reports that THC had minimal effects on LPS-induced IL-8 secretion in THP-1 cells [53]. Thus, the present study suggests that THC could exert its anti-inflammatory effects in part through cytokine, but not chemokine pathways. However, further studies are needed to validate this hypothesis.

For medicinal use, CBD and THC can be administered through various routes, including inhalation, oral ingestion, sublingual application, and topical/transdermal delivery systems. The bioavailability, pharmacokinetics, time course, and efficacy of cannabinoids differ depending on the administration method [54]. It has been shown that the therapeutic efficacy of orally administered cannabinoids is reduced by extensive hepatic first-pass metabolism, which is responsible for more than 95% of cannabinoid inactivation, and is associated with a slow absorption rate [55]. These limitations can be circumvented by use of topical/transdermal applications of cannabinoid products [56]. During topical/transdermal delivery, CBD or THC would first contact tissue keratinocytes; for example, if applied as a moisturizing lotion or spray. Thus, delivery of appropriate concentrations of CBD and THC could be critical for controlling tissue disorders, including inflammation. The present study suggests a range of concentrations that could be used as starting points for investigations of topical/transdermal delivery of therapeutic cannabinoids [57]. Future studies that include 3-dimensional gingival and skin tissues as well as animal models need to be performed in order to identify the optimal range of CBD and THC, as well as the delivery levels required to control tissue inflammation [34,58,59].

## 5. Conclusions

The present study demonstrates that at low concentrations, cannabinoids (CBD and THC) are not cytotoxic to gingival and skin keratinocytes, and are not associated with a decrease in cell viability, cell migration, or elevated LDH activity. At concentrations over 20 µg/mL, both CBD and THC are cytotoxic to keratinocytes. Furthermore, high concentrations of CBD and THC can deregulate keratinocyte mitochondrial activity. The present study suggests that cannabinoids (CBD or THC) should be used at concentrations below 20 µg/mL to avoid cytotoxicity. These lower concentrations were able to prevent LPS-induced inflammation by decreasing pro-inflammatory cytokines, including IL-6 and IL-8. Overall, results from this study suggest that CBD and THC may be used in different formulations (e.g., as a moisturizing lotion or spray) in order to manage tissue inflammation caused by pathological conditions, such as lichen planus, dermatitis, and psoriasis.

## Data Availability

The original contributions presented in this study are included in the article. Further inquiries can be directed to the corresponding author.

## References

[1] ElSohly M.A., Majumdar C.G., Chandra S., Radwan M.M. (2024). A 10-year trend in cannabis potency (2013–2022) in different geographical regions of the United States of America. Front. Public Health.

[2] Radwan M.M., Chandra S., Gul S., ElSohly M.A. (2021). Cannabinoids, phenolics, terpenes and alkaloids of cannabis. Molecules.

[3] Elkins A.C., Deseo M.A., Rochfort S., Ezernieks V., Spangenberg G. (2019). Development of a validated method for the qualitative and quantitative analysis of cannabinoids in plant biomass and medicinal cannabis resin extracts obtained by super-critical fluid extraction. J. Chromatogr. B Anal. Technol. Biomed. Life Sci..

[4] Shehata I., Hashim A., Elsaeidy A., Nair A., Urits I., Viswanath O., Kaye A.D., Habib M. (2022). Cannabinoids and Their Role in Chronic Pain Treatment: Current Concepts and a Comprehensive Review. Health Psychol. Res..

[5] Thapa D., Patil M., Warne L.N., Carlessi R., Falasca M. (2025). Enhancing Tetrahydrocannabinol’s Therapeutic Efficacy in Inflammatory Bowel Disease: The Roles of Cannabidiol and the Cannabinoid 1 Receptor Allosteric Modulator ZCZ011. Pharmaceuticals.

[6] Chester L.A., Englund A., Chesney E., Oliver D., Wilson J., Sovi S., Dickens A.M., Oresic M., Linderman T., Hodsoll J. (2024). Effects of Cannabidiol and Delta-9-Tetrahydrocannabinol on Plasma Endocannabinoid Levels in Healthy Volunteers: A Randomized Double-Blind Four-Arm Crossover Study. Cannabis Cannabinoid Res..

[7] Deutsch D.G. (2016). A Personal Retrospective: Elevating Anandamide (AEA) by Targeting Fatty Acid Amide Hydrolase (FAAH) and the Fatty Acid Binding Proteins (FABPs). Front. Pharmacol..

[8] Pertwee R.G. (2008). The diverse CB1 and CB2 receptor pharmacology of three plant cannabinoids: Delta9-tetrahydrocannabinol, cannabidiol and delta9-tetrahydrocannabivarin. Br. J. Pharmacol..

[9] ALSalamat H.A., Abuarab S.F., Salamah H.M., Ishqair A.H., Dwikat M.F., Nourelden A.Z., Qandil A.N., Barakat Y., Barakat M. (2024). Cannabis and cancer: Unveiling the potential of a green ally in breast, colorectal, and prostate cancer. J. Cannabis Res..

[10] Motwani M.P., Bennett F., Norris P.C., Maini A.A., George M.J., Newson J., Henderson A., Hobbs A.J., Tepper M., White B. (2018). Potent Anti-Inflammatory and Pro-Resolving Effects of Anabasum in a Human Model of Self-Resolving Acute Inflammation. Clin. Pharmacol. Ther..

[11] Monteiro Viana J.C., da Silva Gomes G.E., Duarte Oliveira F.J., Marques de Araújo L.N., Teles G., Mourão C.F., de Vasconcelos Gurgel B.C. (2024). The Role of Different Types of Cannabinoids in Periodontal Disease: An Integrative Review. Pharmaceutics.

[12] Chaoul N., Palazzo S., Cinquantasei A., Aresta V., De Chirico C., Albanesi M. (2024). Cannabidiol modulation of immune cell function: In vitro insights and therapeutic implications for atopic dermatitis. Postepy Dermatol. Alergol..

[13] Paudel K.S., Hammell D.C., Agu R.U., Valiveti S., Stinchcomb A.L. (2010). Cannabidiol bioavailability after nasal and transdermal application: Effect of permeation enhancers. Drug Dev. Ind. Pharm..

[14] Scheau C., Badarau I.A., Mihai L.G., Scheau A.E., Costache D.O., Constantin C., Calina D., Caruntu C., Costache R.S., Caruntu A. (2020). Cannabinoids in the Pathophysiology of Skin Inflammation. Molecules.

[15] Sunda F., Arowolo A. (2020). A molecular basis for the anti-inflammatory and anti-fibrosis properties of cannabidiol. FASEB J..

[16] Henshaw F.R., Dewsbury L.S., Lim C.K., Steiner G.Z. (2021). The Effects of Cannabinoids on Pro- and Anti-Inflammatory Cytokines: A Systematic Review of In Vivo Studies. Cannabis Cannabinoid Res..

[17] Knapp A.A., Lee D.C., Borodovsky J.T., Auty S.G., Gabrielli J., Budney A.J. (2019). Emerging Trends in Cannabis Administration Among Adolescent Cannabis Users. J. Adolesc. Health.

[18] Rouabhia M., Piché M., Hazzi C., Corriveau M.N., Chakir J. (2023). Effect of cannabis smoke condensate on human nasal epithelial cell adhesion, growth, and migration. Am. J. Otolaryngol..

[19] Boukamp P., Petrussevska R.T., Breitkreutz D., Hornung J., Markham A., Fusenig N.E. (1988). Normal keratinization in a spontaneously immortalized aneuploid human keratinocyte cell line. J. Cell Biol..

[20] Gilchrist E.P., Moyer M.P., Shillitoe E.J., Clare N., Murrah V.A. (2000). Establishment of a human polyclonal oral epithelial cell line. Oral Surg. Oral Med. Oral Pathol. Oral Radiol. Endod..

[21] Ligasová A., Vydržalová M., Buriánová R., Brůčková L., Večeřová R., Janošťáková A., Koberna K. (2019). A New Sensitive Method for the Detection of Mycoplasmas Using Fluorescence Microscopy. Cells.

[22] Bahraminia M., Cui S., Zhang Z., Semlali A., Le Roux É., Giroux K.A., Lajoie C., Béland F., Rouabhia M. (2025). Effect of cannabidiol (CBD), a cannabis plant derivative, against *Candida albicans* growth and biofilm formation. Can. J. Microbiol..

[23] Gaffal E., Cron M., Glodde N., Tüting T. (2013). Anti-inflammatory activity of topical THC in DNFB-mediated mouse allergic contact dermatitis independent of CB1 and CB2 receptors. Allergy.

[24] Kozela E., Juknat A., Kaushansky N., Rimmerman N., Ben-Nun A., Vogel Z. (2013). Cannabinoids decrease the th17 inflammatory autoimmune phenotype. J. Neuroimmune Pharmacol..

[25] Sermet S., Finn B.M., Crawford R.B., Kaminski N.E. (2025). Δ^9^-Tetrahydrocannabinol and cannabidiol selectively suppress toll-like receptor (TLR) 7- and TLR8-mediated interleukin-1β production by human CD16^+^ monocytes by inhibiting its post-translational maturation. J. Pharmacol. Exp. Ther..

[26] Viereckl M.J., Krutsinger K., Apawu A., Gu J., Cardona B., Barratt D., Han Y. (2022). Cannabidiol and Cannabigerol Inhibit Cholangiocarcinoma Growth In Vitro via Divergent Cell Death Pathways. Biomolecules.

[27] Abedin-Do A., Zhang Z., Douville Y., Méthot M., Rouabhia M. (2021). Effect of Electrical Stimulation on Diabetic Human Skin Fibroblast Growth and the Secretion of Cytokines and Growth Factors Involved in Wound Healing. Biology.

[28] Denizot F., Lang R. (1986). Rapid colorimetric assay for cell growth and survival. Modifications to the tetrazolium dye procedure giving improved sensitivity and reliability. J. Immunol. Methods.

[29] Rouabhia M., Rouabhia D., Park H.J., Giasson L., Zhang Z. (2017). Effect of soft foods on primary human gingival epithelial cell growth and the wound healing process. Food Res. Int..

[30] Alanazi H., Rouabhia M. (2022). Effect of e-cigarette aerosol on gingival mucosa structure and proinflammatory cytokine response. Toxicol. Rep..

[31] Kumar P., Nagarajan A., Uchil P.D. (2018). Analysis of Cell Viability by the Lactate Dehydrogenase Assay. Cold Spring Harb. Protoc..

[32] Grobs Y., Romanet C., Lemay S.E., Bourgeois A., Voisine P., Theberge C., Sauvaget M., Breuils-Bonnet S., Martineau S., El Kabbout R. (2024). ATP citrate lyase drives vascular remodeling in systemic and pulmonary vascular diseases through metabolic and epigenetic changes. Sci. Transl. Med..

[33] Jaber S.M., Yadava N., Polster B.M. (2020). Mapping mitochondrial respiratory chain deficiencies by respirometry: Beyond the Mito Stress Test. Exp. Neurol..

[34] Mazzantini C., El Bourji Z., Parisio C., Davolio P.L., Cocchi A., Pellegrini-Giampietro D.E., Landucci E. (2024). Anti-Inflammatory Properties of Cannabidiol and Beta-Caryophyllene Alone or Combined in an In Vitro Inflammation Model. Pharmaceuticals.

[35] Derradjia A., Alanazi H., Park H.J., Djeribi R., Semlali A., Rouabhia M. (2016). α-tocopherol decreases interleukin-1β and -6 and increases human β-defensin-1 and -2 secretion in human gingival fibroblasts stimulated with *Porphyromonas gingivalis* lipopolysaccharide. J. Periodontal Res..

[36] Chen H., Liu Y., Yu S., Li C., Gao B., Zhou X. (2023). Cannabidiol attenuates periodontal inflammation through inhibiting TLR4/NF-κB pathway. J. Periodontal Res..

[37] Christy S., Carlsson A.H., Larson D., Davenport G.J., Glenn J.F., Brumfield Avina G., Jockheck-Clark A., Christy R.J., Nuutila K. (2024). Topical Noneuphoric Phytocannabinoid Elixir 14 Reduces Inflammation and Mitigates Burn Progression. J. Surg. Res..

[38] Kim H.J., Kim B., Park B.M. (2015). Topical cannabinoid receptor 1 agonist attenuates the cutaneous inflammatory responses in oxazolone-induced atopic dermatitis model. Int. J. Dermatol..

[39] Sangiovanni E., Fumagalli M., Pacchetti B., Piazza S., Magnavacca A., Khalilpour S., Melzi G., Martinelli G., Dell’Agli M. (2019). *Cannabis sativa* L. extract and cannabidiol inhibit in vitro mediators of skin inflammation and wound injury. Phytother. Res..

[40] McCartney D., Kevin R.C., Suraev A.S., Sahinovic A., Doohan P.T., Bedoya-Pérez M.A., Grunstein R.R., Hoyos C.M., McGregor I.S. (2023). How long does a single oral dose of cannabidiol persist in plasma? Findings from three clinical trials. Drug Test. Anal..

[41] Spiera R., Hummers L., Chung L., Frech T.M., Domsic R., Hsu V., Furst D.E., Gordon J., Mayes M., Simms R. (2020). Safety and Efficacy of Lenabasum in a Phase II, Randomized, Placebo-Controlled Trial in Adults with Systemic Sclerosis. Arthritis Rheumatol..

[42] Billi M., Pagano S., Pancrazi G.L., Valenti C., Bruscoli S., Di Michele A., Febo M., Grignani F., Marinucci L. (2025). DNA damage and cell death in human oral squamous cell carcinoma cells: The potential biological effects of cannabidiol. Arch. Oral Biol..

[43] Trivedi M.K., Gangwar M., Mondal S.C., Jana S. (2017). Protective effects of tetrahydrocurcumin (THC) on fibroblast and melanoma cell lines in vitro: It’s implication for wound healing. J. Food Sci. Technol..

[44] Lee J.H., Yoon J.Y., Kim D.H., Kwon Y.G., Kim G.H., Park B.J., Suh D.H. (2024). Potential of cannabidiol as acne and acne scar treatment: Novel insights into molecular pathways of pathophysiological factors. Arch. Dermatol. Res..

[45] Tassaneesuwan N., Khongkow M., Jansrinual S., Khongkow P. (2025). Discovering the Potential of Cannabidiol for Cosmeceutical Development at the Cellular Level. Pharmaceuticals.

[46] Diaz P., Katz T., Langleben A., Rabinovitch B., Lewis E. (2021). Healing of a chronic pressure injury in a patient treated with medical cannabis for pain and sleep improvement: A case report. Wound Manag. Prev..

[47] Almada M., Alves P., Fonseca B.M., Carvalho F., Queirós C.R., Gaspar H., Amaral C., Teixeira N.A., Correia-da-Silva G. (2020). Synthetic cannabinoids JWH-018, JWH-122, UR-144 and the phytocannabinoid THC activate apoptosis in placental cells. Toxicol. Lett..

[48] Marzęda P., Wróblewska-Łuczka P., Drozd M., Florek-Łuszczki M., Załuska-Ogryzek K., Łuszczki J.J. (2022). Cannabidiol Interacts Antagonistically with Cisplatin and Additively with Mitoxantrone in Various Melanoma Cell Lines—An Isobolographic Analysis. Int. J. Mol. Sci..

[49] Walker O.S., Gurm H., Sharma R., Verma N., May L.L., Raha S. (2021). Delta-9-tetrahydrocannabinol inhibits invasion of HTR8/SVneo human extravillous trophoblast cells and negatively impacts mitochondrial function. Sci. Rep..

[50] Raouf N., Darwish Z.E., Ramadan O., Barakat H.S., Elbanna S.A., Essawy M.M. (2024). The anticancer potential of tetrahydrocurcumin-phytosomes against oral carcinoma progression. BMC Oral Health.

[51] Chan J.Z., Duncan R.E. (2021). Regulatory Effects of Cannabidiol on Mitochondrial Functions: A Review. Cells.

[52] Jiang Z., Jin S., Fan X., Cao K., Liu Y., Wang X., Ma Y., Xiang L. (2022). Cannabidiol Inhibits Inflammation Induced by *Cutibacterium acnes*-Derived Extracellular Vesicles via Activation of CB2 Receptor in Keratinocytes. J. Inflamm. Res..

[53] Zaiachuk M., Suryavanshi S.V., Pryimak N., Kovalchuk I., Kovalchuk O. (2023). The Anti-Inflammatory Effects of *Cannabis sativa* Extracts on LPS-Induced Cytokines Release in Human Macrophages. Molecules.

[54] Bruni N., Della Pepa C., Oliaro-Bosso S., Pessione E., Gastaldi D., Dosio F. (2018). Cannabinoid Delivery Systems for Pain and Inflammation Treatment. Molecules.

[55] Ohlsson A., Lindgren J.E., Andersson S., Agurell S., Gillespie H., Hollister L.E. (1986). Single-dose kinetics of deuterium-labelled cannabidiol in man after smoking and intravenous administration. Biomed. Environ. Mass. Spectrom..

[56] Valiveti S., Hammell D.C., Earles D.C., Stinchcomb A.L. (2004). Transdermal delivery of the synthetic cannabinoid WIN 55,212-2: In vitro/in vivo correlation. Pharm. Res..

[57] Sivadasan D., Madkhali O.A. (2024). The Design Features, Quality by Design Approach, Characterization, Therapeutic Applications, and Clinical Considerations of Transdermal Drug Delivery Systems-A Comprehensive Review. Pharmaceuticals.

[58] Park J.Y., Lee H.J., Han E.T., Han J.H., Park W.S., Kwon Y.S., Chun W. (2023). 3,4,5-Trihydroxycinnamic acid suppresses phorbol-12-myristate-13-acetate and A23187-induced mast cell activation in RBL-2H3 cells. Exp. Ther. Med..

[59] Suryavanshi S.V., Zaiachuk M., Pryimak N., Kovalchuk I., Kovalchuk O. (2022). Cannabinoids Alleviate the LPS-Induced Cytokine Storm via Attenuating NLRP3 Inflammasome Signaling and TYK2-Mediated STAT3 Signaling Pathways In Vitro. Cells.

